# Old drugs, new weapons: current trends in repurposing therapies against antimicrobial resistance

**DOI:** 10.3389/jpps.2026.16158

**Published:** 2026-06-05

**Authors:** Gatadi Srikanth, Niggula Praveen Kumar, Bhima Sridevi, Padma Bhavani Borra, Parul Thapar, Aabid Wani

**Affiliations:** 1 GITAM School of Pharmacy, GITAM (Deemed to be University), Hyderabad, India; 2 Department of Pharmaceutical Chemistry, Bharat Institute of Technology, Hyderabad, India; 3 Department of Pharmacy, KL College of Pharmacy, Koneru Lakshmaiah Education Foundation (Deemed to be University), Vaddeswaram, Guntur, India; 4 Department of Pharmaceutical Chemistry, Vision College of Pharmaceutical Sciences and Research, Hyderabad, Telangana, India; 5 Department of Food and Nutrition, Swami Vivekananda University, Barrackpore, West Bengal, India

**Keywords:** adjuvants, antimicrobial resistance, combination therapy, drug repurposing, pathogens

## Abstract

Antimicrobial resistance (AMR) is a globally accelerating issue threatening the efficacy of existing treatments. Repurposing approved drugs for the development of new antimicrobial agents reduces the development cost and risk. The clinical potential of repurposed medications for AMR is essential to implement coordinated strategies that encompass scientific validation and supportive regulatory frameworks. Repurposing serves as a strong method to prolong the effectiveness of current antimicrobial classes and to fill significant voids in the global AMR pipeline. The present review summarizes current trends in repurposing strategies against AMR, utilization of non-antibiotic drugs with antibacterial activity, agents that potentiate conventional antibiotics through membrane disruption or efflux pump inhibition, and host-directed therapies that modulate immune responses and combination therapies. We also highlight advances in systems pharmacology, *in silico* screening, and phenotypic assays that enable rational identification of repurposing candidates. Although significant regulatory and economic barriers persist, including weak intellectual property protection, limited commercial incentives, and market constraints, swift attempts are being made to address the issues.

## Introduction

Antimicrobial drugs are a vital class of medications that treat infections and enable complex medical procedures, like cancer treatments, organ transplants, and surgeries, to be performed infection-free [1]. The emergence and spread of multi drug-resistant bacteria affect the safety of critical treatments like organ transplants, hip replacements, cancer chemotherapy, and caesarean deliveries [2]. Antimicrobial resistance (AMR) is the ability of microorganisms to render antimicrobial agents ineffective, which leads to long persistent infections that cause increased morbidity and mortality [3]. Overuse of antimicrobials, poor infection control, and improper sanitation and hygiene all contribute to AMR.

AMR affects individuals of all ages around the world. Infections caused by resistant pathogens, often referred to as “superbugs,” are becoming increasingly difficult and, in some cases, impossible to manage. Pathogens that are prone to antimicrobial resistance, including the ESKAPE group such as *Staphylococcus aureus*, *Pseudomonas aeruginosa*, *Enterococcus* faecium, *Klebsiella pneumoniae*, *Acinetobacter* baumannii, and *Enterobacter* spp., as well as carbapenem-resistant Enterobacteriaceae (including ESBL-producers), drug-resistant *Mycobacterium tuberculosis* (MDR/XDR TB), and drug-resistant *Neisseria* gonorrhoeae—along with ESBL-producing *E. coli* and K. pneumoniae—pose critically significant threats due to their ability to evade standard antimicrobial treatments, thereby restricting global treatment options [4].

Current treatments for AMR infections face significant constraints. Many older classes of antibiotics no longer work against a wider range of MDR and XDR pathogens. Even the “last-line” drugs like Colistin and Tigecycline have become less effective for MDR/XDR against Gram-negative infections [5]. Both treatments are still linked to high rates of death and treatment failure [6]. Thus, MDR infections are linked to high death rates and long hospital stays and often require combination therapy, which can be unsuccessful [7]. This situation shows how important it is to find new antimicrobial agents, better ways to diagnose infections, and better ways to manage AMR.

### The global challenge of antimicrobial resistance

AMR has emerged as a significant threat to global health. Bacteria have become stronger at resisting the medications that are supposed to kill them. The WHO estimates that antibiotic resistance is highest in the South-East Asian and Eastern Mediterranean regions, where 1 in 3 reported infections were resistant. In the African region, 1 in 5 infections was resistant. Resistance is also more common and worsening in places where health systems lack the capacity to diagnose or treat bacterial pathogens. Bacterial AMR contributed to an estimated 4.95 million fatalities in 2019, with a concerning 1.27 million of the fatalities being directly caused by infections that were resistant to treatment [8]. The burden is not evenly shared; low- and middle-income countries (LMICs) suffer the brunt of the impact as their health systems have been less effective, their diagnostic tools are limited, and they have more risk factors, including lack of good nutrition and immunocompromised patients. Many factors contribute to AMR, such as the overuse and misuse of antibiotics in human medicine and veterinary and agricultural settings, poor infection prevention and control (IPC) practices, inadequate diagnostics and surveillance, and the spread of resistant organisms beyond borders via travel, trade, and the environment [9]. Clinically, the outcome is extended hospitalizations and heightened morbidity and mortality, especially for children, the elderly, and the immunocompromised. If AMR is not effectively addressed, it may necessitate substantial economic investment [10-12]. Some studies say that LMICs could lose more than 5% of their GDP by 2050, and the world could lose up to $100 trillion. Clinically, this leads to longer hospital admissions and higher rates of illness and death, especially in children, the elderly, and people with weak immune systems [13]. If we do not take quick, coordinated action involving better stewardship, global surveillance, more money for diagnostics, and the creation of novel therapies, the stealthy spread of resistance may undo decades of progress in treating infectious diseases and render the health system more susceptible overall [14].

### The concept of drug repurposing: definition and rationale

**FIGURE 1:**
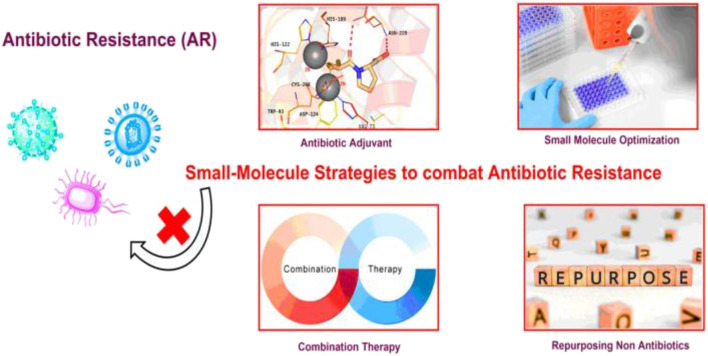
Small molecule strategies to overcome Antibiotic Resistance (AR).

Drug repurposing, also known as drug repositioning, is a good alternative for (or addition to) conventional *de novo* drug development, with the aim of finding newer applications outside the scope of the original medical indication for existing drugs that are currently on the market or have already received approval [15]. Repurposing makes extensive use of pre-existing safety and clinical data [16]. The benefits of employing this specific approach include cost-effectiveness and less time needed for the drug discovery process. The drug repurposing approach is especially attractive in the context of AMR [17]. A combination of repurposed drugs and conventional antibiotics can be used to fight resistant bacteria such as *S. aureus*, *K. pneumoniae, P. aeruginosa,* and *Escherichia coli* (Figure 1). However, issues like serious off-target effects, toxicity, and intellectual property and regulatory issues [18] pose serious challenges. Because repurposed medications already have the manufacturing and shipping infrastructure, pharmacokinetic and toxicological data, and established safety profiles, they may be a quicker and less expensive option.

In the realm of antimicrobial therapeutics, repurposed drugs may operate as direct bactericidal or bacteriostatic agents, function as adjuvants that augment the effectiveness of current antibiotics (for instance, efflux pump inhibitors or β-lactamase inhibitors), or engage in host-directed mechanisms that alleviate infection or the development of resistance [19]. The approach is promising, but it has some problems, such as less effective potency in new indications, complicated dosing and pharmacology, and possible unintended effects on resistance dynamics [20]. Therefore, repurposing serves as a strategic complement to conventional antibiotic discovery rather than a substitute. From a mechanistic standpoint, repurposing may yield (i) direct antibacterial activity from non-traditional compounds (e.g., non-antibiotic drugs exhibiting off-target antimicrobial effects); (ii) adjuvant effects when used alongside existing antibiotics to restore or enhance efficacy (e.g., efflux-pump inhibitors, quorum-sensing inhibitors, or agents disrupting biofilms); and (iii) host-directed therapies modulating host immunity or pathophysiology rather than directly targeting bacterial viability [21].

### Scope and objectives of the review

This review comprehensively focusses on drug repurposing as an emerging strategy to overcome AMR. The scope includes the study of resistance trends across bacterial pathogens, surveillance data, molecular mechanisms [22], trends and gaps in antibiotic research and development (R&D), drug repurposing strategies, computational platforms, preclinical and clinical investigations, and regulatory considerations. Incorporation of precision medicine approaches, phenotypic screening, combination therapy, and synergistic drug repurposing.

## Historical perspective on antimicrobial resistance

AMR is one of the most critical global public health threats of the 21st century [23]. According to global estimates in 2019, AMR was highest in low- and middle-income countries [24]. High costs with low return incomes, and the challenges of discovering new scaffolds, led the major pharma giants to step back from antibiotic R&D. Over the last four decades, there has been far less development of new antibiotic classes targeting resistant Gram-negative bacteria [25]. This gap highlights an urgent need for alternative therapeutic strategies that can challenge the AMR crisis. A wide range of non-antibiotic drugs like anti-cancer drugs exhibit antimicrobial activity through efflux pump inhibition, membrane disruption, and immune modulation [26]. Computational biology, artificial intelligence (AI), chemogenomics, and systems pharmacology helped drug repurposing to achieve a high success rate [27]. High-throughput screening of drug libraries, AI-based drug–target prediction, network pharmacology models, host-directed therapies (HDTs), and immune-modulating repurposed drugs have accelerated the identification of candidate drugs and further refined therapeutic approaches for persistent infections such as MDR-TB [28]. Overcoming the limitations like dose-dependent toxicity, insufficient antimicrobial potency, and pharmacokinetic constraints limitations require coordinated efforts, such as integrating different disciplines and global policy initiatives [29]. In this context, the present review aims to bridge molecular biology, pharmacology, and computational sciences to highlight repurposing-based strategies as a promising and accelerated route to tackle AMR [30].

### Limitations of traditional antibiotic development

Comparatively simple screening of soil-derived microorganisms and abundant natural-product reservoirs drove the early decades of antibiotic discovery. However, the scientific community established what is now referred to as an antibiotic discovery “void” when these easily accessible sources ran out. As a consequence of growing scientific and translational challenges, the approval of new antibiotic classes has drastically decreased since the 1970s. Establishing compounds that may penetrate bacterial envelopes, especially the impermeable outer membrane of Gram-negative pathogens, minimizing intrinsic resistance determinants, obtaining acceptable pharmacokinetic and safety profiles, and sustaining activity in the face of rapidly evolving resistance are major challenges [31].

Significant financial and regulatory disincentives exacerbate scientific barriers. In comparison with other medications for chronic illnesses, antibiotics have a significantly lower potential for profit because they are usually prescribed for brief periods of time and are increasingly reserved as last-line treatments. Many pharmaceutical companies have withdrawn from antibacterial research and development as a result of this limited market viability. Because of this, there is a growing gap between clinical need and therapeutic availability as antimicrobial resistance continues to rise globally, and the antibiotic innovation pipeline is severely underpowered.

### Advantages of repurposing existing drugs for AMR

Some repurposed drugs show direct antimicrobial activity, some show adjuvant effects, and some exhibit host‐directed therapies, indirectly reducing infection burden. Repurposing is a vital alternative to traditional antibiotic development, as it is an efficient mode of therapy while the R&D of novel antibiotics are still in the pipeline [32].

### Key advantages of repurposing existing drugs for AMR

The primary benefits stem from leveraging the extensive R&D already completed for the original drug, leading to a faster, cheaper, and lower-risk path to new therapies [32] (Table 1).

**TABLE 1:** Advantages and impact on antimicrobial resistance.

Advantage	Description	Impact on AMR
Reduced time to market	The drug has already passed the initial, time-consuming stages of discovery and preclinical testing	Treatments can reach patients with life-threatening resistant infections faster (estimated 3–12 years vs. 10–17 years for a new drug)
Lower development costs	Repurposing largely bypasses expensive early-stage R&D and phase I safety trials	Significantly lower financial investment (estimated $300 million vs. $1.6 - $2 billion for a new molecular entity)
Established safety profile	The safety, toxicity, pharmacokinetics, and pharmacodynamics (ADME) are already well-known from previous clinical trials and/or market use	Reduces the high risk of failure associated with unknown safety in new drugs, leading to higher approval rates
Enhanced Efficacy/Synergy	Repurposed drugs can be used as adjuvants (helpers) to enhance the effectiveness of existing antibiotics	They can inhibit bacterial resistance mechanisms (e.g., efflux pumps, biofilm formation) or work in combination therapy to re-sensitize resistant bacteria
Diverse mechanisms of action	Many existing non-antibiotic drugs (e.g., anti-inflammatories, antifungals, psychiatric drugs) act against bacteria via unique mechanisms	Offers novel ways to attack resistant pathogens, making it harder for bacteria to develop resistance to the new treatment
Simplified regulatory pathway	For food and Drug administration (FDA)-approved drugs, a shorter regulatory pathway may sometimes be used (e.g., 505(b)(2) application), leveraging existing data	Accelerates the path to clinical validation and approval for the new indication

## Overview of resistance mechanisms in bacteria

The prevalence of antimicrobial resistance is high in low-middle income countries that lack access to medical facilities, have poor water sanitation, and have limited medicines and diagnostics. Many diseases caused due to certain microorganisms are difficult to treat. It is regarded as a silent pandemic, posing threats to humans and ecosystems around the world (Global tuberculosis report, 2019). It is estimated that the increased abundance of antimicrobial resistance could lead to loss of 3.8% of global annual GDP. In extreme cases, an additional 24 million people may be forced towards extreme poverty, hunger, and malnutrition if not controlled (World Health Organization report, 2023) [33].

### Resistance in fungi, viruses, and parasites

The microorganisms that develop antimicrobial resistance are referred to as “superbugs”. These may be bacteria, fungi, virus, or protozoa. The resistant bacterial species of resistant Gram-negative bacteria that are pathogenic include *Salmonella*, *Helicobacter pylori*, *Campylobacter jejuni*; *Shigella dysenteriae, Shigella flexneri*, and *Shigella sonnei*. Gram-negative bacteria are found to show intrinsic resistance to multiple drugs. Some Gram-positive bacteria have also been found to be multi-drug resistant and include *Enterococcus faecalis*, *Staphylococcus aureus*, Methicillin-resistant *Staphylococcus epidermidis* (MRSE), and MDR *S. epidermidis*. The genera of fungi that are found to be resistant include Aspergillus, *Candida*, and Pneumocystis [34–40]. The organisms and their resistance to certain antibiotics are mentioned in Table 2.

**TABLE 2:** Microbial species Resistant to Antibiotics.

Microorganisms	Antibiotic resistant species	Antibiotics	References
Bacteria	*Campylobacter jejuni*	Ciprofloxacin, fluoroquinolones, nalidixic acid, tetracycline, ampicillin, erythromycin, gentamicin, and enrofloxacin	[40]
	*Helicobacter pylori*	Amoxicillin, metronidazole, clarithromycin, tetracycline, levofloxacin	[38, 40]
	*Enterococcus faecalis*	Vancomycin and beta-lactam antibiotics	[41, 42]
	*Staphylococcus haemolyticus*	Last-line antibiotics glycopeptides	[37, 43, 44]
	*Escherichia coli*	Third- generation cephalosporins, carbapenems, first-line drugs- ampicillin and co-trimoxazole; second line drugs-fluoroquinolones	[45]
	*Klebsiella pneumoniae*	Carbapenems	[45]
	*Mycobacterium tuberculosis*	Rifampicin and other drugs	[46]
Fungi	*Aspergillus fumigatus*, *Candida tropicalis*, *Candida parapsilosis*, *Candida auris*	Triazoles	[47–49]
	*Pneumocystis*	Sulfa-prophylaxis, trimethoprim-sulfamethoxazole	[50]
Protozoa	*Neisseria gonorrhoeae*	Ciprofloxacin	[45]

The formation of biofilms on the outer membranes of bacteria is one of the major hinderances for the effectiveness of certain antibiotics. A biofilm allows microbes to resist host immune responses and the effect of antibiotics. Both Gram-positive and Gram-negative bacteria can form biofilms, leading to an ongoing battle on various surfaces.

The infection that is caused due to antimicrobial resistance developed through biofilms is termed “biofouling.” It is a polysaccharide-encased multicellular bacterial aggregation adhering to various surfaces. Biofilm infection is recognized to be responsible for more than 65% of infections, posing a huge threat to public health. Biofilm-forming microorganisms possess different characteristics like collective cooperation, resistance to antimicrobials, and source capturing [41–48, 51, 52].

### Role of resistance mechanisms in guiding repurposing strategies

Repurposing strategies are also referred to as repositioning or reprofiling of drugs. The resistance mechanisms in microorganisms have helped to create new formulations for antibiotics that were not able to prevent microbial proliferation during the occurrence of a disease. The process of repurposing is found to be safe during human clinical trials and quite effective.

**FIGURE 2:**
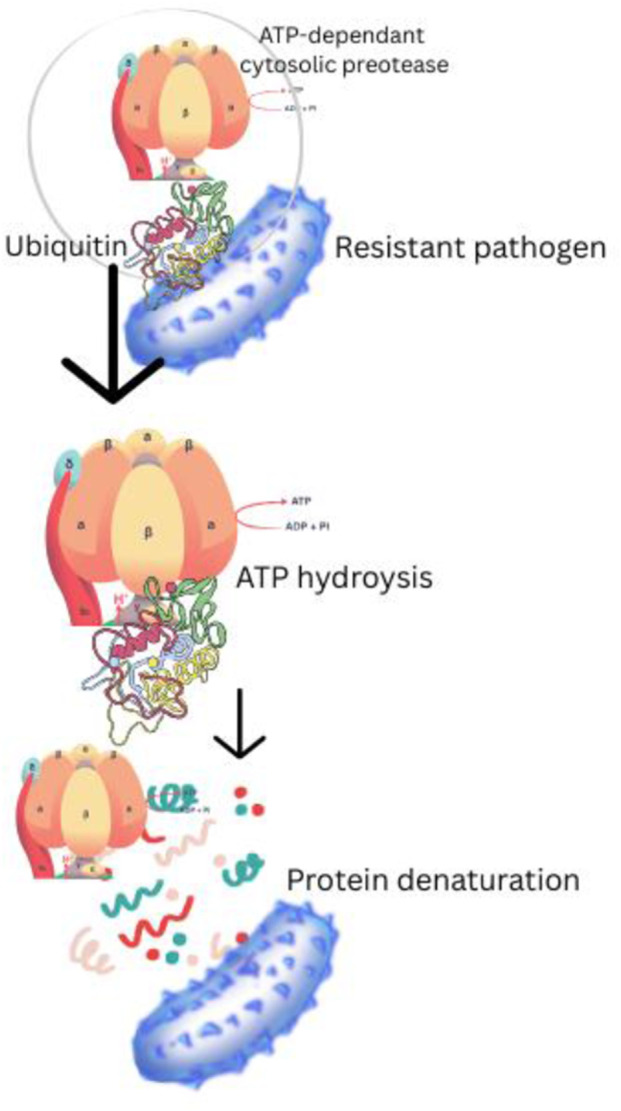
Repurposing Strategies involved in Protein unfolding and overcoming drug resistance in Bacteria.

The repurposing of drugs involves a ubiquitin-proteasome degrading system (UPS). UPS is a major cytosolic protein degradation system. Ubiquitin is an evolutionary conserved protein which can be degraded at 19 S proteasome (bounded ATP dependant cytosolic protease) in resistant bacteria. The process targets 19 S proteasome components-deubiquitinases POH1 and USP14/UCHL5 (D’Arcy et al, 2011) and ubiquitin-binding receptor RPN13 that causes ATP hydrolysis. Also, the repositioning of drugs is done to inactivate ubiquitin activating enzyme (UEA1) by TAK-243 or inhibition of p97/VCP segregase upstream by proteasome CB5083. Both these compounds target the resistance mechanisms, thereby improving the antibiotic entry into the microorganisms. The unfolded proteins along with the drugs are actively translocated into proteolytic chambers in the proteases (Figure 2). It is found that these molecular targets have shown to overcome resistance with bortezomib/carfilzomib antibiotics in resistant microbes [49, 50, 53–66].

## Current trends in drug repurposing for AMR

### Repurposing non-antibiotic drugs

Antibiotic resistance is caused by the inappropriate use of antibiotics and is considered a major global issue, causing significant deaths all over the world. Moreover, with increasing medical expenses and decreasing numbers of approved antimicrobial agents [67], alternative strategies to combat antibiotic resistance is the need of the hour. One such strategy is repurposing non-antibiotic drugs. Drugs that are originally designed for disease mitigation that subsequently possess antimicrobial properties are classified under drug repurposing as “non-antibiotics” [68]. Since COVID-19, therapies using drug repurposing approaches have accelerated and the antimicrobial activity of drugs belonging to different categories like antidepressants, antihypertensives, anti-inflammatories, antineoplastics, and hypoglycaemic agents have been explored thoroughly. In this particular section of the review, current trends in the repurposing of anticancer, antidepressants, antipsychotics, anti-inflammatory, antifungals, and immunomodulatory agents, as well as existing antibiotics for AMR, are discussed.

#### Repurposing of anti-cancer drugs

Cancer patients have a compromised immune system and hence hospital-acquired infection-related complications pose a major threat, especially in the case of AMR. Cancer and bacterial cells behave similarly in their proliferation rate, metabolism, survival, and adaptation techniques, cell-to-cell communication, and drug-resistance mechanisms [69]; therefore, structurally similar anticancer drugs are anticipated to act as anti-microbials to some extent. Drug resistance mechanisms involve drug inactivation, overexpression of efflux pumps (EPs), and drug-target modifications. EPs mediate cell–cell communication and biofilm formation in bacteria, whereas in cancer they promote cancer growth by causing an efflux of anticancer drugs. Drug resistance mechanisms in both bacterial and cancer may be innate or acquired. Several investigations showed that certain anticancer drugs exhibit a direct relationship to antimicrobial activity [70] (Table 3). Anti-cancer drugs like Tocilizumab, Ruxolitinib, and Baricitinib and interferons like IFN-A1B, IFN-A2B, IFN-B1A, IFN-B1B, IFN-B2, Peg-IFN-L1A, Emapalumab, Bevacizumab, Nivolumab, Pembrolizumab, Leronlimab, Imatinib, Thalidomide, Lenalidomide, Acalabrutinib, Ibrutinib, and Selinexor are at various stages of clinical testing for their potential repurposing in the management of COVID-19 [75]. Linsitinib, an experimental potent drug candidate targeting pGlnA1 for the treatment of cancer, has been found to have direct antimycobacterial activity [76].

**TABLE 3:** Some of the anti-cancer drugs repurposed for combating AMR.

S. No	Mechanism of action	Drug candidates for repurposing	Active against strain type
1	Direct inhibition-DNA crosslinking	Cisplatin	*S aureus, P aeruginosa, and E coli*
2	Induce oxidative stress	Etoposide	*P aeruginosa*
3	Targets the production of virulence factors and biofilm in bacteria	5-Fluorouracil [71]	*S. aureus and S. epidermidis*
4	Inhibition of Fe (III) transport, DNA replication	Gallium nitrate	*P aeruginosa, A. baumannii, M. tubrrculosis*
5	DNA crosslinker	Mitomycin C	*Acinetobacter baumannii* [72]
6	Reduced viral RNA-dependent RNA polymerase (RdRp)-mediated gene expression	Aclarubicin	SARS-CoV-2 infection [73]
7	Enhancement of oxidative stress	Elesclomol	Mtb H37Rv [74]
8	Impeded biofilm formation	Ponatinib	*S. mutans*
9	Disruption of bacterial membrane	Floxuridine	*S. suis strains*
10	Bacterial phosphorylation by deoxyribonucleoside kinase	Gemcitabine	*MRSA, MSSA, GISA*

### Existing challenges in repurposing anti-cancer drugs

Repurposing non-antibiotic drugs may pose clinical implications like disrupting immune regulation or gut microbiome imbalance. Since the fundamental design and development of the anti-cancer drugs is to target cell proliferation, these agents pose the risk of impeding immune responses, resulting in secondary infections and many other complications. Therefore, preventing over-suppression and further complications for patients should be considered.

### Antidepressants and antipsychotics

Bacteria reduce antimicrobial drug accumulation through certain specific mechanisms, such as the over expression of bacterial efflux pumps. These bacterial efflux pumps can be inhibited with antidepressants and antipsychotics, which act as efflux pump inhibitors (EPIs) [77]. Selective Serotonin Reuptake Inhibitors (SSRIs), Serotonin and Norepinephrine Reuptake Inhibitors (SNRIs), tricyclic antidepressants (TCAs), and phenothiazine-type antipsychotics are considered as EPIs as they exhibit efflux pump-modulating activity (Table 4). However, persistent use of antidepressant drugs can disrupt the gut bacteria, leading to potent adverse effects. Further, these drugs are required in slightly higher concentrations for antimicrobial effects than the therapeutic concentrations required for psychiatric conditions, raising serious toxicity concerns.

**TABLE 4:** Antidepressants and Antipsychotics repurposed for AMR.

S. No	Mechanism of action	Drug candidates for repurposing	Active against strain type
1	MFS-(NorA and TetK) and RND-class (AcrB) EPI [78]	Paroxetine	*S. aureus*
2	EPI	Sertraline	*S. aureus, E. coli, P. aeruginosa, Helicobacter pylori*
3	Inhibits bacterial biofilm formation, EPI	Fluoxetine	*Proteus mirabilis, S. aureus, E. coli, P. aeruginosa*
4	Inhibits the AcrAB-TolC efflux system	Amitriptyline	*Salmonella Typhimurium*
5	Reversal of plasmid-mediated resistance	Imipramine, desipramine	*E. coli K12 strain*
6	Reduction of the NorA-mediated ethidium bromide efflux	Thioridazine, chlorpromazine, fluphenazine	*S. aureus*

### Adjuvant therapies to enhance antibiotic efficacy

Antimicrobial resistance in microorganisms occurs through several mechanisms: the presence of ß-lactamase enzymes, which disrupt the molecular structure of antibiotics and inactivate them; and the development of efflux pumps, which expel antibiotics to decrease their concentration within the cells. The molecular structure of the target site of antibiotics may be modified through microbial genetic mutations. These mutations also cause altered ribosomal structure, leading to damage in intrinsic enzymatic synthesis that causes reduction in the binding affinity of antibiotics and makes them ineffective [79–85].

Based on the above mechanisms, certain antibiotic adjuvants can play an important role in inhibiting these pathways and contribute to the prevention of antibiotic resistance in microorganisms. Antibiotic adjuvants are the agents or substances that are co-administered with antibiotics that increase the activity of antibiotics. These substances function by improving their effects on a suitable target, resulting in an increase in the antimicrobial effects. They are also involved in the pathways associated with antimicrobial resistance and modulate the host immune response for an overall increase in the therapeutic effects. It was shown that treatment with an antibiotic adjuvant along with an antibiotic has more potential effectiveness in treatment than an antibiotic alone against the infections caused by drug-resistant microorganisms [antibiotic + adjuvant (chemical agent)] [86–89].

The adjuvants lower the minimum inhibitory concentration of the antibiotics and enhance the concentration of already-existing antibiotics (Melander and Melander [90]).

#### Types of antibiotic adjuvants

Depending upon the mechanism of inhibition involved, antibiotic adjuvants can be classified as either Class I or Class II antibiotic adjuvants. Class I adjuvants can be further sub-divided into Class IA and Class IB adjuvants. Class IA adjuvants are called “inhibitors of active resistance”; these are involved directly to inhibit the resistance mechanisms by modifying the activity of ß-lactamase enzyme and efflux pump system and modify the drug targets within the microorganisms. Class IB are referred to as “inhibitors of passive resistance.” They increase the antibiotic effects by modifying the signalling and regulating pathways of antibiotics within the microorganisms. Class II adjuvants are called “host-modulating adjuvants” as they modify the host response by activating the immune response or phagocytic activity of the immune cells. Class II adjuvants still require further exploration [91–95]. The classification of types of adjuvants is given in Table 5.

**TABLE 5:** Classification of Adjuvants with their mechanism of action.

Class	Subclass	Category	Mechanism of action	Examples	Reference
I	IA (active resistance inhibitors)	ß-lactamase inhibitors	Inhibition of serine and metallo- ß-lactamase	Clavulanic acid, Sulbactam, avibactam	[93]
	IB (passive resistance inhibitors)	Efflux pump inhibitors	Inhibit generated efflux pumps (MFS, SMR, MATE, RND etc.)	MC-207, 110 (phenylalanyl arginyl); ß-naphthylamide; CCCP (carbonyl cyanide-m-chlorophenylhydrazone)	[95]
		Biofilm-disrupting agents and membrane permeabilizers	Inhibits the formation of biofilms in microorganism and enhance membrane permeability to increase antibiotic uptake	NAC (N-acetyl cysteine), Tween 80, D-amino acid, norspermidine, DspB (Dispersin B), DNase I and α-amylase Guanidinylated polymyxins, colistin, cerium oxide nanoparticles	[92, 96]
II	Host defence mechanism	Immune enhancers	Modulates host immune system and phagocytic response	Antimicrobial peptides, active vitamin D, phenylbutyrate, human cathelicidin	[81, 90]

### Beta-lactamase inhibitors

**FIGURE 3:**
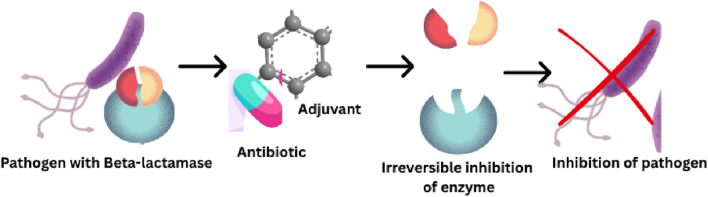
Inhibition of pathogen by ß-lactamase inhibitor (Antibiotic and adjuvant).

Class IA (active resistance inhibitors) can be combined with ß-lactam antibiotics including penicillin, cephalosporins, carbapenems, and monobactams. These antibiotics consist of a ß-lactam ring that is antimicrobial in nature. When these antibiotics are administered alone, the ß-lactamase enzyme within the microbes degrade this ring by opening its molecular structure, thereby making it ineffective against the microorganism. When combined with an inhibitor, it causes an irreversible inhibition of ß-lactamase enzyme, thereby dissociating its activity. This retains the efficacy of the antibiotic against the pathogen. An example would be augmentin [combination of clavulanic acid (ß-lactamase inhibitor) and amoxicillin (antibiotic)], which causes irreversible inhibition of the ser- ß-lactamase (serine ß-lactamase) enzyme (Figure 3). These inhibitors possess broad spectrum inhibition against enzymes like extended spectrum ß-lactamase (ESBL) and carbapenemases [96–100]. Other examples are mentioned in Table 2.

### Efflux pump inhibitors

Efflux pumps are cellular transport proteins that are found within pathogens. These molecules are utilized to remove the antibiotics by reducing their intracellular concentration and efficiency. This leads to antibiotic resistance within the pathogens. The efflux pumps found in only Gram negative bacteria include the resistance nodulation and cell division family; those found within both Gram positive and Gram negative bacteria includes ABC-ATP binding cassette; the SMR family of small multi drug resistance; the MFS major facilitator superfamily; the MATE multidrug and toxin extrusion family; and the PACE proteobacterial antimicrobial compound efflux superfamily. The inhibition in the activity of antibiotic transport by efflux pumps could be processed by Efflux Pump Inhibitors (EPI) [101–105].

**FIGURE 4:**
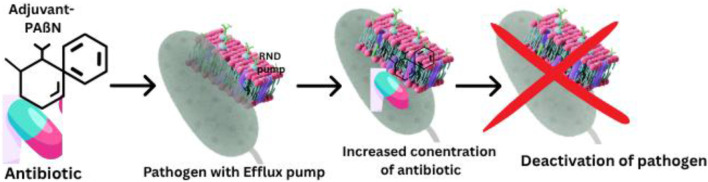
Deactivation of a pathogen by EPI.

EPI are class IB (passive resistance inhibitors) which, when administered with antibiotics, usually increase the intracellular concentration of antibiotics within the pathogens. When EPI are used as adjuvants in combination with antibiotics, they prevent the removal of the antibiotic from the pathogen, thereby improving the antibiotic efficiency. Examples of EPI used are PAßN- phenylalanine-arginine-ß-naphthylamide and CCCP carbonyl cyanide m-chlorophenylhydrazone in combination with antibiotics such as levofloxacin or erythromycin (Figure 4). Their antimicrobial effects have been shown against strains of *Pseudomonas aeruginosa* (Table 6).

**TABLE 6:** Suitable Examples of Antibiotic adjuvants.

Category	Adjuvants	Antibiotics combined	References
ß-lactamase inhibitor	Diazabicyclooctanones (DBOs) (non ß-lactam)	Ceftazidime, imipenem and cilastatin	[106]
	Vaborobactam	Meropenem	[104, 107]
	Tazobactam	Piperacillin, ceftolozane	[104, 107, 108]
	Sulbactam	Ampicillin	
	Avibactam	Ceftazidime	
Efflux pump inhibitor	PAßN- phenylalanine-arginine ß-naphthylamide	Levofloxacin or erythromycin	[103]
Biofilm disrupting agents	Chitosan	Streptomycin	[109]

### Biofilm-disrupting agents and membrane permeabilizers

**FIGURE 5:**
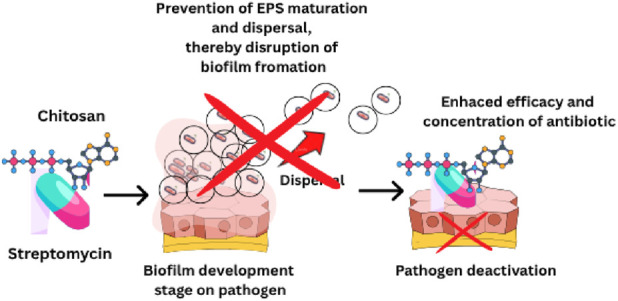
Prevention of EPS maturation and dispersal, thereby disrupting biofilm formation.

Biofilms are defined as communities of properly organised microorganisms attached to a substrate and embedded in a self-produced extracellular matrix (extracellular polymeric substances - EPS), which aids cell interactions. It is a sequential phenomenon that depends on a plethora of factors (physical, chemical, and biological) in its different phases: attachment, microcolony formation, maturation, and dispersion. During these phases, which may vary a little in their complex pattern, microbial biofilms usually form three-dimensional structures separated by porous water channels and cavities. These channels and cavities allow nutrient entrance and waste discharge. The polysaccharides that comprise the EPS form the skeleton of the biofilm, providing structural stability. The 3D architecture of a biofilm can change its thickness, shape, and structure through time, but this evolution does not necessarily result in progressive orders. In fact, biofilms are dynamic structures whose evolution depends on both biotic and abiotic factors acting on time scales ranging from seconds to years. These biofilms have an anaerobic environment which is not favoured by antibiotics. Due to the formation of these biofilms, some species of Gram-positive and Gram-negative bacteria are developing resistance to antibiotics. There are certain enzyme and chemical agent adjuvants that may play an important role in the disruption of these biofilms by disturbing the phases of biofilm formation and improving the permeability of membranes. Through this, there occurs enhancement in the penetration of an antibiotic within the bacteria and increased antibiotic efficiency. The adjuvants that can prevent the maturation of EPS during biofilm formation include specific agents like enzymes like NAC (N-acetyl cysteine), Tween 80, D-amino acid, norspermidine, DspB (Dispersin B), polyamine and enzymes-glycosyl hydrolase, DNase I, and α-amylase (Figure 5) [106–110].

Along with these adjuvants, there are certain membrane permeabilizers which, when consumed together, help in improved localization and concentration of antibiotics within the bacteria. These include natural compounds like thymol, gallic acid, and glycosylated cationic block poly beta peptide (PAS8-b-PDM12), antimicrobial peptides such as colistin and detergents and nanoparticles such as PMBN (polymyxin B nanopeptide) and cerium oxide nanoparticles chitosan derivatives- 2,6-DAC (2,6- diamino chitosan), glycine-containing amino acid-conjugated polymer (ACP), polyurethanes, and polycarbonates (Table 6) [111–119].

### Immunomodulatory agents and anti-inflammatory agents

The development of drug resistance amongst common bacterial pathogens has led to an alarming increase in immunocompromised patients with impaired immune function. Immunomodulatory therapies act by targeting the immune response modulations of the host rather than the pathogen. This is an alternative approach for the treatment of infectious diseases caused by anti-microbial resist pathogens [120]. Antimicrobial peptides (AMPs) are immunomodulators that have applications in targeted therapy, wound-healing, and drug delivery systems. Synergistic application of AMP-based strategies with current antibiotics might reduce drug load and provide improved results [121]. Mycograb, a human recombinant antibody fragment, combined with amphotericin B showed activity against resistant *Candida* species [122].

Dual action drugs with dual mechanistic approaches are the need of the hour in order to minimise multi drug administration, drug-drug interactions, and other adverse effects. The presence of multi-drug resistance efflux pump systems is why Gram-negative bacteria show higher resistance against anti-inflammatory drugs. Synergistic action of NSAIDs and antibiotics effectively combats the emergence of multi-drug resistance in microorganisms. Those NSAIDs that control the generation of pro-inflammatory mediators are considered as effective for treating infections. The following Table 7 lists the anti-inflammatory drugs repurposed for AMR and Table 8 lists the other non-antibiotic classes repurposed for AMR.

**TABLE 7:** Anti-inflammatory drugs repurposed for AMR.

Anti-inflammatory drug for repurposing	Mechanism of action	Active against strain type
Auranofin	Inhibits the bacterial thioredoxin reductase enzyme [123]	VISA, VRSA, MRSA
Aspirin + cefuroxime	Anti-bio film effect [124]	MSSA, MRSA
Diclofenac	Reduction in biofilm formation [125] Inhibition of staphyloxanthin synthesis [126]	MRSA
Celecoxib	Inhibition of the synthesis of RNA, DNA, protein	VISA, VRSA, MRSA
Ibuprofen	Membrane disruption, anti-bio film effects	*P. aeruginosa, B. cereus*, *E. coli*, MRSA and MSSA
Carprofen	Inhibition of mycobacterial drug efflux; restriction of mycobacterial biofilm growth [127]	*M. tuberculosis*
Diflunisal	Inhibition of AgrA-mediated gene expression; limits the ability of *S. aureus* to destroy osteoblasts [128]	MRSA

**TABLE 8:** Other non-antibiotic classes repurposed for AMR.

Mechanism of action	Other drugs repurposed	Active against strain type
ETBR blockers	Macitentan (endothelin receptor antagonist)	Human Cytomegalovirus infection
Cytotoxic [129]	Atorvastatin and rosuvastatin (HMG-CoA reductase inhibitor)	*E. coli, P. aeruginosa, S. pneumoniae*
Disruption of *H. pylori* proton motive force [130]	Niclosamide (anthelmintic)	*S. aureus* [131]*, H. pylori 60190 ATCC 49503* [132]
Reversible inhibitor of mitochondrial NADH dehydrogenase [133]	Metformin (anti-diabetic)	*E. faecalis ATCC 29212*
NA^a^	Ivacaftor (CFTR potentiator)	MRSA, MSSA, VRSA [134]

^a^ Information not available.

### Repurposing existing antibiotics and antifungals

Widespread overuse and misuse of antibiotics has led to antimicrobial resistance and hence repurposing existing or older antibiotics has become increasingly important. Repurposing antibiotics eliminates selective strains and therefore causes co-resistance to other antibiotics which possess anti-microbial resistance [135]. Repurposed antibiotics are administered in higher dosages, which triggers multi-drug resistance. Levofloxacin and doxycycline are being repurposed with the aim to treat Alzheimer’s and malaria, respectively, although without satisfactory results. However, the optimisation or redevelopment of older and less explored antibiotics which have high potency and maintained efficacy = against microorganisms could help to tackle AMR in a strategic way.

### Rediscovering older antibiotics

The reintroduction of colistin and fosfomycin helped fight resistant pathogens causing neonatal sepsis [136]. The fosfomycin disodium formulation targeted key elements of the bacterial metabolism and thus showed virulence against fosfomycin-resistant bacterial strains. *In vitro* studies of a PEGylated colistin prodrug developed by Chongyu Zhu et al., showed similar or better antimicrobial performance against multi drug resistant isolates of *Pseudomonas aeruginosa* and *Acinetobacter* baumannii [137]. *In vitro* and *in vivo* studies of minocycline, an old broad-spectrum semisynthetic tetracycline antibiotic, proved its activity against MDR *Acinetobacter* species [138]. Ciclopirox, an anti-fungal agent with a unique mode of action affecting bacterial metabolism, showed activity against multidrug-resistant pathogens [139].

### Combination therapies with repurposed antibiotics

Monotherapy is no longer a promising strategy to treat AMR infections. The development of newer drugs involves cost input as well as time. Adopting combination therapies provides options to circumvent the substantial investment required for the development of new drugs. Combination therapy enhances the potency of drugs with low dosages. However, it also poses serious challenges when compared to monotherapy. Drug-drug interactions leading to adverse reactions is a common problem with combination therapy. Synergistic action with improved drug efficacy to suppress pathogen resistance and simultaneous reduction of drug toxicity towards the host cells is a major requirement of combination therapy [140].

#### Repurposed antibiotic-antibiotic combination therapies

Antibiotic-antibiotic combination therapy follows different mechanisms. Each antibiotic in the combination inhibits different targets in its own pathway. For example, the combination therapy of anti-tubercular drugs such as rifampicin, isoniazid, ethambutol, and pyrazinamide works synergistically: rifampicin inhibits RNA synthesis by targeting DNA-dependent RNA polymerase, isoniazid blocks mycolic acid synthesis essential for cell wall formation, ethambutol interferes with arabinogalactan synthesis in the cell wall, and pyrazinamide disrupts membrane energetics and intracellular survival. Together, this multi-targeted approach enhances bactericidal efficacy, shortens treatment duration, and significantly reduces the emergence of drug resistance. The combination of sulfamethoxazole and trimethoprim inhibits different targets in the same pathway. Streptogramins and pristinamycins inhibit the same target in different pathways [141]. Nonetheless, antibiotic-antibiotic combination therapies are less used because of their intrinsic resistance mechanisms, unpredictable toxicity, and associated pharmacokinetic and pharmacodynamic problems. Restoration of combinations into clinical use which overcome the above challenges would help in combating AMR.

#### Repurposed antibiotic- non-antibiotic combination therapies

Combining an antibiotic with a non-antibiotic compound enhances the activity of the antibiotic. It helps in occluding the antibiotic resistance and delays the onset of resistance mechanisms in the pathogen. One important drawback for this approach is the possibility of drug-drug interactions. A combination of broad-spectrum antibiotics with FDA-approved non-antibiotics helps in fighting infections caused by extremely drug-resistant pathogens [142]. Table 9 shows the list of antibiotic-non-antibiotic combination therapies repurposed for AMR.

**TABLE 9:** Antibiotic-non-antibiotic combination therapies repurposed for AMR.

Antibiotic	Antibiotic/adjuvant	Mechanism	Active against
Tunicamycin	Oxacillin	TarO inhibitor	MRSA
Fosfomycin	β-lactam antibiotic	Cell wall synthesis inhibitor	MRSA
Amoxicillin	Augmentin	Cell wall biosynthesis inhibitor	MRSA
INH	Vit D3+PBA	Activation of autophagy	*M. tuberculosis*

## High-throughput screening and computational approaches in drug repurposing

In modern drug discovery, techniques like high-throughput screening (HTS) and computer-based techniques have a crucial role in the fields of biotechnology, particle science, and enzyme engineering. HTS accelerates the screening of data compounds on various targets within a short span of time, while computational tools such as molecular docking, QSAR predictions, Machine learning (ML) models, and molecular simulations support the analysis and interpretation of these data sets. Concurrently they promote the discovery process by lowering the cost and timelines, thus enhancing the accuracy of scientific investigation.

HTS has emerged as a result of the use of a sophisticated robotic system that rapidly screens chemical compound libraries using microplates.

- Automation: In this process, miniature robotic arms are employed to dispense and automate tasks like incubation and pipetting samples. These tasks are automated and data is recorded [143].
- Miniaturization: Synthetic reactions are carried out in micro well plates (96, 384 etc.) with minimum volume to check the feasibility of the process, thereby reducing the capital and resources usage [144].
- Parallel tasks: More processes are performed simultaneously, providing conclusions for a large number of experiments within a short time.
- Standard Optimization: Designs are optimized to decrease the variability in experiments and enhance precision among multiple experiments.
- Integration: Coordination among all the steps is carried out by an automated robotic system.
- Assembling real-time data: These systems are equipped with sensors and image capture mechanisms for accurate and continuous time monitoring and information collection.
- Scalability: Systems are designed to optimize the reaction conditions form pilot plant to bulk scale production [145].

Alongside micro-well formats, quantitative HTS (qHTS) has taken the lead in creating concentration-based response curves for various compounds, accelerating pharmacological profiling and rapid SAR predictions. Microfluidic droplet screening techniques at small volumes (e.g.: 200,000 drops per second) have decreased the usage of expensive chemicals. Three-dimensional cellular assays like spheroids and organoids have emerged as physiologically relevant techniques with respect to HTS. Proper mathematical validation is a prerequisite for HTS assay. Metrics like Z-factor (Z-prime) compute test quality before forwarding to full assay. A Z-factor >0.5 usually represents strong deviations among positive and negative controls. High-throughput screening (HTS) has expanded beyond small-molecule libraries; to investigate gene functions in the cancer environment, pooled perturbations are performed using CRISPR–Cas9 knockout screens, enabling systematic identification of genes that regulate tumor growth, survival, and response to therapy. HTS along with the microfluidic approach supports the detection of major mutant libraries to design genotype-phenotype terrains efficiently [146].

Virtual screening plays a crucial role in HTS-based silico approaches. This screening involves the testing of millions of molecules by molecular docking into a single known target protein and evaluating their binding scores. Other methods such as QSAR studies, pharmacophore modelling, and structure-based drug design further enriches the lead molecule prior to *in vitro* and animal testing. These techniques usually reduce experimental techniques, cutting costs and time by filtering probable high-binding lead molecules from the large chemical library [147].

### HTS in drug repurposing

Classical HTS performs the rapid identification of effective compounds from a vast compound library. For instance, many antibiotic compounds that displayed greater than 90% inhibition against Trypanosoma cruzi and Methicillin resistant *Staphylococcus aureus* (MRSA) have been identified from ReFRAME libraries [148].

High-content assays like DNA Encoded Chemical Libraries (DECLs) and cell painting technologies make phenotype-based repositioning more valuable than other techniques. DECL allows ligand-protein selection at unprecedented levels. Cell painting, meanwhile, enables the detection and reading of high-resolution images while recording treatment-based morphological changes. High-throughput LC/MS, NMR, FIA-MS, and MALDI-FT-MS and related techniques assist in rapid filtration of hits with undesirable pharmacology and safety profiles and thereby support early ADME/toxicity prediction [149].

### Computer approaches in repurposing

Computer-based screening quickens the evaluation process of repurposing compounds from large known compound libraries. For instance, structure-based docking modules (such as AutoDock and Glide) prioritize docking scores, free enthalpy, and stability by incorporating drug-likeness properties and molecular dynamics simulations. During recent SARS-CoV-2 HTS screening, only three promising leads were identified after docking two million ligand-protein associations by applying filters such as drug likeliness and molecular dynamics [150]. Repurposing strategies based on networking enables link prediction and graph-containing algorithms by creating networks among target-drug, disease-drug, or target-target associations. The Heterogene Graph Transfer Module (HGTDR) can assimilate various drugs, proteins, and disorders to estimate new interactions in end-to-end drug discovery cycles.

Overall, experimental HTS methods and cutting edge *in silico* methods (particularly molecular docking, Pharmacophore/QSAR approaches, Network mapping, and Machine learning) have aided the drug repositioning process by quickly evaluating large libraries, estimating therapeutically useful drug-protein interactions, and establishing mechanistic approaches. These hybrid explorations promise faster therapeutic innovations.

## *In vitro* and *in vivo* screening techniques

### *In vitro* screening assays

#### Phenotypic cell-based assays

These assays employ disease-specific *in vitro* cell lines to determine the effectiveness of the drug compounds on growth, alteration in morphological characteristics and signalling pathways, and cellular endpoints. 96-/384- micro well plates are utilized to test already-approved drug libraries for repurposing molecules. High-content screening (HCS) enhances the quantification of various phenotypic changes (cytoskeleton, signalling pattern, and protein expression) through automation of microscopy, thus creating assumption-free identification of phenotypic modifications [151].

#### Advanced *in vitro* models

Tissue composition, cell-cell associations, drug responses, and metabolic degradations are efficiently summarized by the spheroids, 3D organoids, iPSC-derived models, and organ-on chip models compared to that of 2D cultures. These models enrich translational importance and speed up toxicity/metabolic testing.

#### Biological and chemical binding assays

Biophysical approaches such as Cellular Thermal Shift Assay (CETSA), thermal-based protein profiling, or binding mass spectrometry analyses drug-protein binding, off target interaction, and new modes of action for repurposing drug candidates. These techniques are crucial to detect drug-target engagement and specificity [152].

#### *In vitro* ADME and toxicology

Metabolism and *in vivo* clearance are predicted by hepatocyte and microsomal enzyme assays while cell toxicity or inflammatory mediator responses are quantified by MTT/MTS, ATP, neutral red, and ELISA. These predictions can guide safety assessment of the repurposed drugs.

### *In vivo* screening methods

#### Tiny complete organism model

Zebrafish larvae support high-throughput imaging-based testing in microwell plates and have been utilized to repurpose FDA-approved drugs for β-cell differentiation, CNS behavioural alterations, and protection against ototoxic antibiotics. *Caenorhabditis elegans* and *Drosophila melanogaster* are models with preserved phenotypic and genetic pathways. They are cost effective, high-throughput *in vivo* models.

#### Mammalian models

Xenograft mouse models are widely employed to assess the cytotoxic capability of repurposed drugs *in vivo*. For example, the anticancer potential of temozolomide in tumor xenografts was established as a result of screening of 182 approved agents. Standard rodents and primate models are crucial to analyse drug efficacy, ADME, and safety in physiological contexts [153].

## Machine learning and artificial intelligence in drug repurposing

AI and ML are advancing drug repurposing through quick validation of large numbers of biological data sets including proteomics, genomics, transcriptomics, clinical data, and real-time data to explore unidentified drug-disease relationships. As repositioned drugs already have prior tested safety profiles, AI enhances the recognition of new pharmacological uses, reducing costs and time enormously compared to *de novo* drug discovery [154].

### Classical AI and ML models

For virtual screening and QSAR modelling studies, supervised ML techniques like support vector Machines (SVM), logistic regression, and Random forest and neural-based networks are successfully utilized. These approaches identify drug-protein interaction or detect compounds with high potential for novel therapeutic effectiveness for repurposing. Deep learning architectures and graph relying methods such as convolutional neural networks (CNNs), recurrent networks (RNs), graph neural networks (GNs), and Generative adversarial networks (GANs) are known to generate Structure activity relationships (SAR), predict drug coordination, standardize molecular entity, and analyse data from different models. Prior trained GNNs and knowledge-based graphic models such as NETTAG and Deep MDS allow polypharmacology forecasting across genetic-drug networks [155]. XAI techniques clarify why a drug is advised for a specific disease, providing increased ability to achieve regulatory acceptance. Approaches like Drug Agent install multi-agent frameworks that correlate different language models, drug-protein binding predictors, and knowledge graph agents aid in the discovery of drug repurposing candidates.

### ML- and AI-based drug repurposing-case studies

By applying a deep learning approach to a library of 39,000 compounds, researchers identified the antibiotic halicin. Previously this drug was not related to antibiotics. The model prioritized the candidates based on their potential to inhibit drug-resistant bacteria: Lab screening displayed potential activity *in vitro* and *in vivo* in mice against resistant microbes like *Acinetobacter baurmannii and C. difficile* [156].

#### Repositioning for rare diseases using clinical AI modules

MATRIX is a knowledge-graph AI trained on vast biomedical interactions. It is used to identify new matches for repurposing over 18000 diseases. It has an enhanced rapid treatment strategy for rare disorders such as Castleman disease and matches analogous candidates like dexamethasone, sirolimus, and Cyclophosphamide based on the available literature.

#### Precision AI and polypharmacology models

Healx’s DGEM, Cyclica’s Ligand Express, and IBM Watson are ML and networking modules employed to integrate biochemical pathways, literature searches, and molecular docking. These modules have explored new usage for drugs like metformin and nelfinavir in pulmonary fibrosis of Fragile X syndrome and developed a synergism approach through transcriptomics and network analysis [157].

#### Multi-omics and COVID-19 repurposing

AI-based modules like NETTAG and graph-based autoencoders cluster various omics data to categorize repurposing molecules. Recent COVID-19 studies established priority drugs that matched with the experimental validation by employing these models [158].

## Structure-based drug design and molecular docking

SBDD designs drugs based on the receptor 3D structure (obtained through X-ray crystallography, cryo-EM, or NMR) or in-silico based designed three-dimensional structures of biological targets to develop small molecules with minimum interactions, such as stereo chemical, electrostatic, and hydrophobic, to obtain specificity and binding potential. SBDD is a cyclic process that begins with the identification of an active site of a target structure, followed by the design and docking of the *in silico* ligand, synthesizing the molecule with a high docking score. After that, biological testing of the lead compound through assay, analysis of the receptor-ligand complex, and altering of the design is performed.

### Structure-based drug design

Analysing the phenomenon by which the small molecules identify and associate with the proteins is of high priority in drug development. SBDD involves the rational use of data derived from macromolecular targets [159]. The main aim is to get ligands with sufficient electronic and stearic properties to achieve good binding. The active site’s environment, including the presence of cleft, cavities, sub-pockets, and electrostatic properties, are thoroughly analysed. Present SBDD approaches involve the design of ligands possessing necessary features to regulate the target molecule. Selective regulation of the target protein with high binding ligands will lead to efficient therapeutic benefit [160].

**FIGURE 6:**
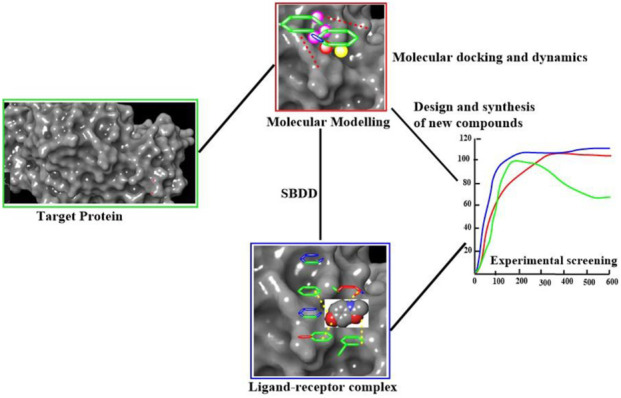
Brief sketch of SBDD. Three-dimensional representation of target protein is used in in silico docking studies. Best score compounds are synthesized and tested by experimental protocols. When the pharmacologically active compounds are discovered, the orientation of the protein receptor complex can be estimated. The binding complex is the basis for discovering new ligands.

SBDD is a sequential process of specific information intake (Figure 6), initiated from a known target structure, followed by identification of active/binding sites, virtual screening and molecular docking of candidate ligands, lead optimization through structure–activity relationships, and subsequent validation using computational and experimental approaches to develop potent and selective therapeutic agents. In silico docking is performed to detect potent small molecules. These molecular modelling techniques are followed by the synthesis of the most potent molecules. After that, the estimation of biological properties such as affinity, binding potency and efficacy are determined by employing various experimental protocols. Provided that highly active molecules are recognized, the three-dimensional structure of Ligand-target complex can be mapped. These structures enable the observations of various intermolecular interactions, supporting the process of molecule recognition. Structural descriptors of ligand-protein complexes assist in establishing binding conformations. Unknown active sites are characterised and ligand-induced conformational changes are explored [161].

#### Basis of docking

The main objective of molecular docking is to predict the protein-ligand complex structure by using docking techniques. Docking can be performed through two mutually important steps. The first step involves predicting the conformation of the small molecule in the active site of the target; then, these conformations are sequenced based on docking scores. Sampling algorithms should be able to match the experimental fitting mode, and scoring function should also align with the generated conformations. These viewpoints build the basics of docking [162].

#### Molecular docking

Molecular docking is considered one of the most useful tools in SBDD because of its capability to investigate the appropriate conformations of a ligand to bind to the target molecule and also serves as an efficient tool to estimate the binding modes and various associations between the molecules that stabilize the drug-protein complex. Molecular docking has gained attention since its emergence in 1980. These algorithms can also emphasize quantitative binding prediction, thereby prioritizing and ranking the molecules based on the binding energy of the ligand-protein complex [163].

The detection of the most effectively binding conformations is done in two stages: (i) uncovering the total conformational space denoting various potential binding poses and (ii) estimating the accurate binding energy linked to each of the binding conformations. The docking technique performs these processes in a cyclical approach, in which the small molecule conformations are assessed based on scoring functions. This process is repeated until the minimum-energy molecule is obtained [164].

#### Conformational search

**FIGURE 7:**
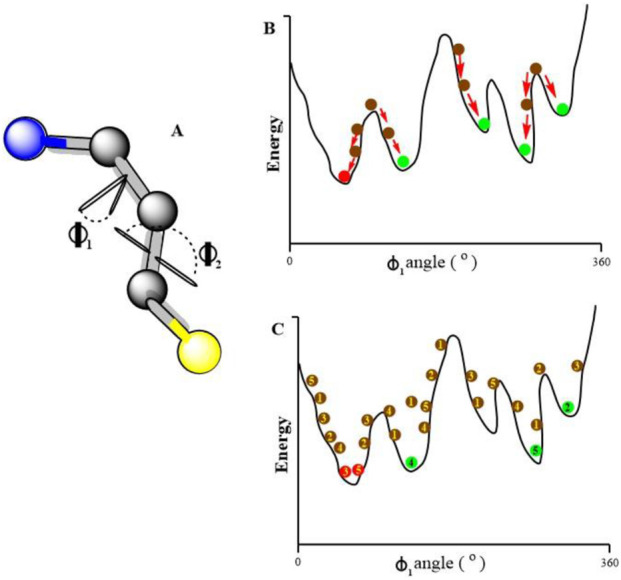
Conformational search methods of a ligand. **(A)** Ligand containing two heavy groups (blue and yellow spheres) has its conformation denoted by two dihedrals Φ_1_ and Φ_2_; **(B)** taking Φ_2_ as a fixed angle, the energy changes due to the rotation of Φ_1_, which is represented in a 1D energy landscape. The initial structure (brown spheres) is altered by changing Φ_1_, resulting in energy reduction. The systemic search algorithm modifies all structural parameters till a local (green spheres) or global (red spheres) energy minimum is attained. **(C)** The stochastic search method reveals the conformational space by randomly creating different conformations.

During the conformational search method, structural parameters of the small molecule, like dihedral/torsion angle and degree of free rotation, are modified (Figure 7A). Conformational search algorithms carry out these modifications by systematic type and stochastic type methods. The systematic method brings structural variation by gradually altering the conformation of the ligand. This algorithm considers energy differences in the conformational space. After performing numerous cycles it converts the minimum energy conformer that denotes the most likely binding pose (Figure 7B). Although this method is efficient, it can converge to the local minima rather than the global minimum. To prevent this, a simultaneous search has to be performed using distinct conformations.

The stochastic method involves a conformational search by random structure modification. To ensure this, the algorithm creates a large group of conformations that represents a wide range of energy barriers (Figure 7C). This approach retards the trapping of a final solution at the local energy minimum and improves the chances of reaching the global minimum. Cost is the major limitation for this process.

Although the specificities of each conformational search algorithm differ, they are capable of uncovering a wide range of energy landscapes within a short span of time. Some of the commonly employed conformational search algorithms are depicted in Table 10 [165].

**TABLE 10:** Commonly used search algorithms.

Stochastic search	Systematic search
AutoDock	FRED
PRO_LEADS	DOCK
Ligand Fit	GLIDE
PLANTS	ADAM
MOE_Dock	Surflex-Dock
Molegro virtual Docker	EUDOC
Gold	SLIDE

#### Estimation of binding energetics

Docking software usually relies on scoring functions to analyse the binding energies of the predicted ligand-protein adduct. The energy changes resulting from complex formation can be attributed to the binding constant (*K*_d_) and Gibbs free energy (Δ *G*_L_). The binding energy is estimated by analysing the physicochemical properties. Therefore, the scoring function will be accurate if more physicochemical properties are considered. As the inclusion parameters increases, the cost also raises; however, if they are too limited the productivity of the docking declines. So ideally there should be a balance between the accuracy, speed, and cost while working.

Scoring functions are differentiated into (i) force field-dependent, (ii) empirical-based, and (iii) knowledge-oriented [166]. Force field type scoring functions predict the binding potential by considering the role of bonded (stretching bending and dihedral difference) and non-bonded interactions (static electric and van der Waals interactions). This function applies an *ab initio* method to predict the energy related to each term using the classical equations. A major limitation of these functions is their inaccuracy in calculating entropic energies [167].

Empirical scoring involves each term of the function denoting an individual physical event that is responsible for the ligand-receptor complex. These usually involve hydrogen bonding, ionic interactions, and apolar interactions along with desolvation and entropic effects. Initially, a series of protein-ligand complexes with known binding efficacies are considered as a training set to carry out analysis through multiple linear regression. Then the weight constants obtained by the mathematical prototype are applied as coefficients that adjust the equation terms. A drawback of these functions is their dependence on the accuracy of the data to build a model [168]. But, due to the usage of simplified energy terms, these functions are rapid and empirical-based. Currently, these functions are mainly used in docking programs like Surflex and FlexX.

Knowledge-based scoring functions utilize pairwise energy potentials obtained from well-known ligand-receptor complexes to deduct a general function. These potentials are built by considering the frequency with which two different atoms are present within a given space in the dataset. Various types of bindings found in the data set are categorized and ranked according to their probability of occurrence. The final score is a combination of these individual interactions. Knowledge-based scoring functions do not depend on empirical methods or *ab initio* calculations; they provide a desirable balance between reliability and quickness [169]. Some of the scoring functions frequently employed in docking programs are presented in Table 11.

**TABLE 11:** Widely used scoring functions in docking programs.

Knowledge-oriented	Force field-dependant	Empirical-based
PMF_Score	GoldScore	F_Score
RF_Score	DOCK	SCORE
Pose score	AutoDock	Fresno
PESD_SVM	SYBYL_D-score	Hyde Ludi
Drug score	SYBYL_G-score	SFC score
MotifScore	LigandFit	PLP
SMoG	ICM	GlideScore
​	​	ChemScore
​	​	AutoDock

## Pharmacokinetic and dynamic considerations in drug repurposing

Although drug repurposing is regarded as a cost-effective and time-saving approach for modern drug dicovery, pharmacokinetic and dynamic properties need to be assessed to ensure safety and effectiveness in the context of a new indication.

### Pharmacokinetic considerations

ADME parameters can vary based on the route of drug intake, target tissue, and also patient group. For example, chloroquine and hydroxychloroquine were initially used as antiplasmodial drugs but were repurposed for COVID-19. However, they exhibited modified pharmacokinetic properties due to extreme tissue binding and long half lives causing cardiac toxicity in some patients [170]. Likewise, antineoplastic tyrosine kinase blockers such as imatinib and nilotinib were repurposed as powerful antivirals. These investigations proved that plasma tissue accumulation levels are acceptable for antiviral potential [171]. Pharmacokinetic challenges are also well established in a case study of remdesivir. Its deprived bioavailabilty demanded intravenous injection of its phospahate ester to attain adequate concentration at the viral replication site. On the other hand, sildenafil, initially introduced for angina, was repurposed for penile dysfunction and later for pulmonary arterial hpertension after kinetic assays uncovered optimal blood concentration and alveolar distribution at medium doses [172].

### Pharmacodynamic considerations

The reinvestigation of pharmacodynamics is to ensure that repositioned drugs provide the desired target at therapeutic doses without causing new adverse actions. Thalidomide could be the best example of this. After being withdrawn due to its teratogenic effects, it was reintroduced as a immunomodulatory drug for multiple myeloma after thorough investigation of its cytokine-regualting PD effects and dose-mediated toxicity [173]. An antidiabetic biguanide, Metformin, has been studied for cancer therapy and age-related disorders, where PD studies explored AMPK-linked antiproliferative activity, without any relation to its anti-diabetic mechanism. Similarly, non-steroidal AntiInflammatory Drugs (NSAIDs), reintroduced as antineoplastic drugs, need to be thoroughly monitored for their dose-activity relationships and cyclooxygenase inhibition patterns to balance therapeutic efficieny against cardiovascular and gastrointestinal toxicity.

### Merging of PK/PD modelling

Drug repurposing approaches mostly depend on PK/PD modelling and physiology-based pharmacokinetic (PBPK) simulations to assess human susceptability, improve dose administration, and optimize translational precision. These models can predict whether current doses can attain sufficient drug exposure at novel target sites or if formualtion and dosing alterations are required. Some of the examples for PK/PD models are presented in Table 12 [174–176].

**TABLE 12:** Repurposed drugs and their employed PK/PD models.

Drug	Original indication	Repurposed indication	Model type	Key PK/PD outcomes
Hydroxychloroquine	Antimalarial	COVID-19	PBPK-Exposure/Response	Lung tissue exposure vs. toxicity
Metformin	Diabetes	Cancer	PBPK + tumor PD	Intratumoral exposure vs. AMPK activation
Imatininb	Chronic myeloid leukaemia (cancer)	Antiviral (SARS/MERS)	Population PK/PD	EC_50_ coverage in plasma/tissue
Remdesivir	Antiviral (ebola)	COVID-19	Two-Compartment PK + metabolite PD	Viral EC_90_ coverage
Sildenafil	Anginal pain	Pulmonary Hypertension	E max PK/PD model	Dose-response for vasodilation

## Case studies of successful repurposing

Examples shown in Table 13 are the molecules that were repurposed for anti-infective chemotherapy. This inbound repurposing of infectious-disease drugs mainly started from cancer chemotherapy. This can be explained from the fact that tumor cells and parasites have a lot in common: i) a fast propagation, which necessitates a high rate of cell division and nucleotide synthesis; ii) causes a faster metabolic activity; and iii) generates radicals (Table 14). Thus, antifolates are used for the treatment of cancers, bacterial infections, and parasitosis. Antifolates are drugs that hamper the synthesis of dTMP from dUMP, where folate acts as the methyl group donor [1–10]. Another common target is pyrimidine synthesis, where dihydroorotate dehydrogenase (DHODH) is being pursued in antimalarial and anticancer drug development. Also, proteasome inhibitors show chemotherapeutical potential against tumour cells as well as parasites [177–186]. Tables 13–16 show successful examples of drug repurposing [187–215].

**TABLE 13:** Inbound repurposing of drugs to the field of infectious diseases.

Molecule	Original purpose	Envisaged new purpose
Cisplatin	Cancer	Antibacterial
Eflornithine	Cancer	Human African trypanosomiasis
Gallium nitrate	Cancer	*Pseudomonas aeruginosa*
Miltefosine	Cancer	Leishmaniasis
Mitomycin C	Cancer	Microbial persisters
Niclosamide antibacterial	Snail control	Antiviral, antifungal
Thalidomide	Morning sickness	Soporific Leprosy
Toremifene	Cancer	Bacterial biofilm

**TABLE 14:** Similarities between parasites and tumor cells and how they can be exploited as drug targets.

Shared Property	Emerging Target/MoA	Drug
High rate of cell division	Microtubule spindle	Mebendazol
Fast rate of DNA synthesis	Topoisomerase	Quinolones
High demand of nucleotides	Pyrimidine synthesis antifolates	DHODH inhibitors
High rate of protein turnover	Proteasome	Proteasome inhibitors
High metabolic rate	Glycolysis	Antimycin A
Enhanced redox metabolism	Activation of prodrugs	Artemisinin
Cellular signalling pathways	Protein kinases	Sunitinib

**TABLE 15:** Drug repurposing within the field of infectious diseases.

Molecule	Original purpose envisaged	New purpose
Albendazole	Veterinary helminthoses	Human helminthoses
Amphotericin B	Antifungal	Leishmaniasis
Artemether	Malaria	Schistosomiasis
Clindamycin	Antibacterial	Malaria
Closantel	Anthelmintic	MRSA
Doxycycline filariasis	Antibacterial	Malaria
Fosmidomycin	Antibacterial	Malaria
Ivermectin human	Veterinary helminthoses	Helminthoses
Levamisole	Anthelmintic	Colon cancer
Nifurtimox	Chagas disease	HAT
Paromomycin	Antibacterial	Leishmaniasis
Pentamidine	African trypanosomiasis	Balamuthiasis, leishmaniasis
Posaconazole	Antifungal	Chagas disease
Sulphonamides	Antibacterial	Malaria
Suramin onchocerciasis	African trypanosomiasis	Antiviral
Telacebec ulcer	Tuberculosis Buruli	Leprosy

**TABLE 16:** Outbound repurposing of drugs from the field of infectious diseases.

Molecule envisaged	Original purpose	New purpose
Allopurinol	Leishmaniasis	Gout
Artemisinin	Malaria	Cancer
Eflornithine	African trypanosomiasis	Hirsutism
Ivermectin	Anthelminthic	Mosquito control
Minocycline	Antibacterial	Neurodegenerative disorders
Pentamidine	African trypanosomiasis	Cancer
Suramin	African trypanosomiasis	Cancer, snake bite, and autism

Anticancer drugs like taxol and vinblastine are active against malaria parasites and etoposide, methotrexate, and doxorubicin have been found to be active against trypanosomatids. However, the therapeutic potential of anticancer drugs are not the same as those of anti-infectives; they differ in particular regarding the final product’s cost-of-goods and safety and that risks and adverse reactions that may be acceptable during cancer treatment are not for diseases such as malaria. The molecules mentioned in Table 3 have been repurposed within the field of anti-infective chemotherapy, from one pathogen to another. This is preferred if the two pathogens are genetically close. A common scenario is the repurposing of veterinary drugs for human medicine to treat infections caused by parasitic nematodes. Indeed, all the drugs currently for human use have actually been developed as veterinary anthelminthics. Drug repurposing has also been found to be possible between phylogenetically aloof pathogens. Antifungals, for instance, can be repurposed against Leishmania and Trypanosoma because both fungi as well as trypanosomatids, have ergosterol and not cholesterol as the sterol component of their membranes [216–225].

Screening of the NIH library for approved drugs against various pathogens has recognized toremifene and clomiphene (selective oestrogen receptor modulators) as potent inhibitors of Zaire ebolavirus infection [226]. The effect appeared not to be related with oestrogen signalling, but rather with an off-target effect where the molecules interfere with viral entry and likely affect the triggering of fusion. A second example is the inhibition of Zika virus propagation, and of virus-induced cytopathic effects, in glial cell lines and human astrocytes by the macrolide antibiotic azithromycin. The antiarthritic metallodrug auranofin exhibits potent macrofilaricidal activity and antileishmanial activity through the inhibition of the redox enzymes. Some phenotypic screenings were customized for a particular therapeutic potential, such as the screening of compounds known to permeate the blood–brain barrier, aiming to identify potential treatments for late-stage HAT, when the trypanosomes have invaded the patient’s cerebrospinal fluid. Moreover, opportunistic repurposing has also been considered for disease-targeted collections. For instance, the Malaria Box is a collection of compounds assembled by the Medicines for Malaria Venture (MMV) to support drug discovery against malaria. While the primary focus obviously is malaria, some of the compounds have also been screened against other pathogens to explore their potential as broad-spectrum antiparasitic agents. This has returned potent hits against kinetoplastids, toxoplasmosis, amoebiasis, candidiasis, schistosomiasis, echinococcosis, and several other diseases [227–236].

The best-known case is the use of the antimalarials chloroquine and hydroxychloroquine in the treatment of COVID-19-associated pneumonia. Studies have demonstrated that chloroquine exhibited significant efficacy in suppressing viral replication, displaying an effective concentration (EC90) value of 6.90 μM. This was a case of rational repurposing based on chloroquine’s established mechanism of action that involves obstructing virus infection through the elevation of endosomal pH and the disruption of the glycosylation of the cellular receptor for SARS-CoV-2. It is also speculated that the immunomodulatory properties of the drug could augment the antiviral effect *in vivo*. In contrast, the outcome of clinical trials was controversial. Many large randomized controlled trials showed no mortality benefit of chloroquine or hydroxychloroquine for hospitalized COVID-19 patients. Finally, in June 2020, the FDA revoked the authorization for emergency use of chloroquine and hydroxychloroquine to treat COVID-19 patients. Probably the oldest antiparasitic drug still in use today, suramin, demonstrated inhibition of SARS-CoV-2 replication and possibly acts on early steps of the replication cycle, preventing the binding or entry of the virus. Suramin was shown to be a potent inhibitor of the SARS-CoV-2 RNA-dependent RNA polymerase (RdRp), blocking the binding of RNA to the enzyme, and was found to be 20-fold more potent than remdesivir. Suramin had been shown to inhibit RNA-dependent DNA polymerase (reverse transcriptase) in 1979. Clinical studies to support the effectiveness of suramin against COVID-19 are lacking. Nitazoxanide, an antiparasitic drug for diarrhoea and enteritis triggered by Cryptosporidium spp. and G. intestinalis of known antiviral activity, inhibited SARS-CoV-2 replication in Vero E6 cells at a low micromolar concentration. However, the resolution of symptoms in patients did not differ between nitazoxanide- and placebo-treated groups after 5 days of therapy. The antiparasitic drug ivermectin is an additional example of the COVID-19 repurposing efforts. The antiviral potential of ivermectin had been recognized in an opportunistic repurposing screen of randomly selected bio-actives for the inhibition of importin αβ-mediated nuclear import. Ivermectin was subsequently shown to inhibit the replication of HIV and Dengue virus, and it also inhibited SARSCoV-2 replication in cell cultures. However, clinical studies revealed that treatment with ivermectin did not result in a lower incidence of hospital admissions or of prolonged emergency department observation among outpatients with an early diagnosis of COVID-19. The repurposing attempts for COVID-19 underline the importance of making sure, before going to clinical trials, that not only the activity and tolerability of a drug candidate match the new TPP but also its pharmacokinetics and pharmacodynamics [237–245].

## Challenges and limitations

While drug repurposing is an advantageous approach, the process still faces some challenges. Firstly, using known drugs implies the need to evaluate related intellectual properties, which may potentially hamper the ability to patent repurposed drugs. Other major challenges concern prices and sales. The lack of patentability reduces profit opportunities, discouraging pharmaceutical companies from pursuing this strategy. Initiatives aimed at facilitating drug repurposing through streamlining the marketing authorization process and allocating dedicated funds have been proposed. However, re-patenting a known drug is possible only if its therapeutic activity identified by repurposing is unknown. Yet many of the potential uses found by pharmaceutical repositioning may already be documented in the literature and in clinical practice. Secondly, access to inherent pharmacovigilance and clinical trial data in therapeutic repositioning has long been limited by commercial and confidentiality issues. To overcome this limitation, the EMA made all clinical trial data public from October 2016. Moreover, if a higher dosage of the repositioned drug is required, or if a different route of administration is used than the original, it may be necessary to undergo phase I clinical trials, thereby increasing costs. Remarkably, seven government-run programs in the United States, European Union, and United Kingdom have recently been established to help organizations reduce costs or mitigate one or more of these drug repurposing challenges [246–266].

## Regulatory and economic barriers

### Intellectual property and patent issues

Drug repurposing for AMR often relies on compounds that are off-patent or near expiry, which creates weak intellectual property protection and limited commercial exclusivity for developers. Unlike novel chemical entities, repurposed agents rarely qualify for strong composition-of-matter patents; instead, protection is typically restricted to new-use, formulation, or combination patents, which are narrower and more easily challenged. This weakens a company’s ability to secure returns on investment in costly Phase II–III clinical trials [267].

Extra complexity ascends from fragmented compulsory licensing rules, global patent laws, and varying data exclusivity periods, which together create uncertainty around market access and freedom-to-operate. In a few cases, the original patent holder may still retain control over proprietary clinical data or manufacturing rights, which can complicate collaboration and licensing. For non-antibiotic drugs used as host-directed therapies or adjuvants, the off-label prescribing can further erode incentives to obtain formal AMR indications. Harmonizing incentives for novelty with public-health obligations—particularly for life-saving antimicrobials—is a challenge. Without better-quality IP frameworks, such as transferable exclusiveness vouchers, public-sector investments and incentives, extended data exclusivity, or stewardship-based rewards, the development pipeline for repurposed AMR therapies may continue to lag behind clinical need [268].

### Funding and market incentives

The financial situation for AMR therapeutics is exceptionally constrained. Repurposed drugs often require considerable new investment for clinical authentication, pharmacokinetic studies, formulation optimization, and regulatory approval—yet the expected economic return is comparatively low. Stewardship policies that confine antimicrobial use (appropriately) limit sales volume potential even for successful products.

Outdated “sales-based” market models therefore fail to incentivize investment in AMR-focused repurposing, low-price legacy drugs, or generic drugs. Small and theoretical inventers struggle to attract venture capital, while large pharmaceutical companies regularly prioritize higher-return beneficial areas. Fragmented payer policies, recompense uncertainty, and limited accessibility of push-funding mechanisms add further risk. To overcome these barriers, advanced financing and incentive models are progressively supported. These comprise subscription-style expenditures (“Netflix models”), market entrance rewards, public–private partnerships, milestone-based grants, and not-for-profit development models. Positioning regulatory pathways with these incentives, while ensuring reasonable global access, will be critical to translating hopeful repurposed candidates into widely deployable AMR treatments [269].

## Future directions and opportunities

Unconventional computational approaches, which include artificial intelligence (AI) and machine learning (ML), are gradually being applied to speed up both repurposing efforts and drug discovery [270, 271]. These computational tools make it possible to inspect massive datasets generated from proteomics genomics, chemo-informatics, and clinical research to reveal repurposing opportunities and estimate drug-target interactions. Using the data-driven algorithms, ML and AI systems can estimate similarities between drugs, target proteins, gene expression patterns, and diseases’ chemical structures, thereby supporting the identification of capable candidates. These methods also contribute to ranking drug leads, suggesting new drug combinations, refining dosage strategies, and treating infectious disease [272]. During the COVID-19 pandemic, ML played a significant role in hastening repurposing research. Recently, deep learning approaches contributed to the finding of abaucin, a new antibiotic with strong and selective activity against the Gram-negative pathogen *Acinetobacter baumannii*. In general, ML and AI are altering the landscape of drug repurposing for infectious diseases by increasing data understanding, streamlining optimization processes, and improving predictive accuracy [273]. Their capacity to swiftly pinpoint potential therapeutic solutions is particularly valuable during public health emergencies and for boosting the efficiency of modern drug development pipelines [274–278].

## Conclusion

Antimicrobial resistance has appeared as a defining global health challenge, decreasing the effectiveness of current antibiotics and outpacing the growth of new antimicrobial classes. In this manner, drug repurposing provides a practical and augmented strategy to expand the therapeutic arsenal against multidrug-resistant pathogens [279, 280]. By leveraging current safety, the repurposed drugs, whether functioning as adjuvants, direct antimicrobials, or host-directed therapies, can address urgent clinical needs. Advances in computational methods for evaluating biology, artificial intelligence, phenotypic screening, and systems pharmacology have further changed repurposing from an unregulated approach into a systematic discovery pathway. However, the conversion of promising repurposed agents into routine clinical practice remains inhibited by key scientific, supervisory, and economic barriers. Factors such as achieving effective therapeutic concentrations, formulations, and optimal dosing, mitigating toxicity risks, and navigating feeble intellectual-property protections limit clinical uptake and commercial incentives [281–284]. Coordinated global efforts are essential to link laboratory science, clinical validation, sustained financial investment, and regulatory reform. Public or private partnerships, subscription-based compensation, strengthened surveillance, data-sharing frameworks, and stewardship systems will be crucial in revealing the full potential of repurposed drugs. Eventually, drug repurposing should be seen not as a replacement for novel antibiotic discovery but as a powerful complementary strategy that can alleviate the current therapeutic situation while innovation rebuilds [285–289].
